# Identification of Virulence Factors in Entomopathogenic *Aspergillus flavus* Isolated from Naturally Infected *Rhipicephalus microplus*

**DOI:** 10.3390/microorganisms11082107

**Published:** 2023-08-18

**Authors:** Cesar A. Arreguin-Perez, Estefan Miranda-Miranda, Jorge Luis Folch-Mallol, Raquel Cossío-Bayúgar

**Affiliations:** 1 Centro Nacional de Investigación Disciplinaria en Salud Animal e Inocuidad, Instituto Nacional de Investigaciones Forestales Agrícolas y Pecuarias INIFAP, Boulevard Cuauhnahuac 8534, Jiutepec 62574, Morelos, Mexico; cesaraarreguinp@gmail.com (C.A.A.-P.); miranda.estefhan@inifap.gob.mx (E.M.-M.); 2 Centro de Investigación en Biotecnología, Universidad Autónoma del Estado de Morelos, Av. Universidad 1001, Cuernavaca 62209, Morelos, Mexico; jordi@uaem.mx

**Keywords:** cattle tick, bioassay, morphotype, aflatoxin, kojic acid, chitinases, ribotoxin

## Abstract

*Aspergillus flavus* has been found to be an effective entomopathogenic fungus for various arthropods, including ticks. In particular, natural fungal infections in cattle ticks show promise for biocontrol of the *Rhipicephalus* (*Boophilus*) *microplus* tick, which is a major ectoparasite affecting cattle worldwide. Our study aimed to elucidate the specific entomopathogenic virulence factors encoded in the genome of an *A. flavus* strain isolated from naturally infected cattle ticks. We performed morphological and biochemical phenotyping alongside complete genome sequencing, which revealed that the isolated fungus was *A. flavus* related to the L morphotype, capable of producing a range of gene-coded entomopathogenic virulence factors, including ribotoxin, aflatoxin, kojic acid, chitinases, killer toxin, and satratoxin. To evaluate the efficacy of this *A. flavus* strain against ticks, we conducted experimental bioassays using healthy engorged female ticks. A morbidity rate of 90% was observed, starting at a concentration of 10^5^ conidia/mL. At a concentration of 10^7^ conidia/mL, we observed a 50% mortality rate and a 21.5% inhibition of oviposition. The highest levels of hatch inhibition (30.8%) and estimated reproduction inhibition (34.64%) were achieved at a concentration of 10^8^ conidia/mL. Furthermore, the tick larval progeny that hatched from the infected tick egg masses showed evident symptoms of *Aspergillus* infection after incubation.

## 1. Introduction

*Rhipicephalus microplus* is the most important ectoparasite for the bovine cattle industry in tropical and subtropical cattle-grazing areas around the world [1]. During the cattle tick’s life cycle, it is able to develop from nonparasitic stages of eggs and larvae in the soil [2]; the parasitic stages develop entirely on a single *Bos* genus bovine host [3]. Cattle ticks inflict direct damage on bovines, including skin lesions, anemia, irritability, weight loss, immunosuppression, and reduced milk yield [4]. Additionally, during the parasitic stages, *R. microplus* may transmit bovine infectious diseases such as anaplasmosis and babesiosis [3], causing additional economic losses in bovine production by increasing abortions, veterinary care costs, and fatalities [4,5,6].

*Aspergillus flavus* is a saprophytic fungus that feeds on plant and animal debris [7]. It is also the most common *Aspergillus* species, infecting several naturally occurring species of arthropods [8]. The majority of *A. flavus* strains reported in the scientific literature are capable of producing aflatoxins that can cause aflatoxicosis and may infect immunocompromised humans [9]. *A. flavus* also shows phytopathogenic properties and can infect and reduce the yield of different economically important crops, such as corn, rice, cotton and peanuts [10,11,12]. In vitro laboratory cultures of *A. flavus* exhibit two morphotypes defined by sclerotia size; those with sclerotia over 400 µm in diameter are considered morphotype L, whereas those with sclerotia below 400 µm in diameter are considered morphotype S [13]. In addition to the sclerotia size difference, morphotype S is usually toxigenic, producing B1, B2, G1, and G2 aflatoxins, whereas morphotype L can vary from being atoxigenic to toxigenic and is not capable of synthesizing all types of aflatoxins. [13,14,15,16]. Furthermore, the morphotype is associated with niche adaptation, with the L morphotype being more likely to be found on crops such as maize, and the S morphotype being more likely to be found in soils with surface temperatures near 30 °C [17]. Additionally, S morphotype *A. flavus* genomes are over one Mbp larger, coding over one hundred more predicted genes than the L morphotype [15]. *A. flavus* has been demonstrated to exhibit acaropathogenic properties against different tick species such as *R. microplus* [18,19], *Hyalomma dromedarii* [20], *Amblyomma maculatum* [21], and *Dermacentor albipictus* [22]. In a previous study, it was found that *A. flavus* infected *R. microplus* and produced aflatoxin B1, G1, and G2. This strain also exhibited an experimental mortality rate of 64 ± 19% among engorged females, indicating its potential as a control agent. Additionally, it demonstrated ovicidal potential by successfully colonizing 80% of the ovigerous masses of 24 ticks, resulting in a low hatching rate of only 13%. Natural fungal infection was observed from June to October, most likely due to the relative humidity [18,19]. Unfortunately, the original strain was lost, underscoring the need to isolate a new strain for further analysis of the tick control potential of *A. flavus*. It is worth noting that *A. flavus* is considered a viable alternative for biocontrol during the free-living stages of various tick pests, as it poses minimal mycotoxicity risk to the human food supply [18,20].

### Aspergillus sp. Virulence Factors 

Several species of *Aspergillus* are capable of producing a variety of virulence factors that can damage arthropods during fungal infections. These virulence factors may include ribotoxins, expressed as extracellular proteins capable of inhibiting protein synthesis by cleaving the conserved sarcin-ricin loop of the larger rRNA at the ribosomes, a crucial step for protein expression, leading to systemic cell death by apoptosis [23,24,25]. Hirsutellin A is a ribotoxin protein from the aphid fungal parasite *Hirsutella thompsonii* that is capable of killing several species of insect larvae, in vitro-cultured insect cells, and several species of mites [24], including the phytophagic citrus rust mite *Phyllocoptruta oleivora* [26]. Previous analysis of the *A. flavus* genome revealed that ribotoxin ortholog genes are present in this fungal species and should be considered a possible entomopathogen invasive factor [27].

Chitinases are enzymes that break down chitin, the second most abundant natural polymer after cellulose and a major component of the exoskeleton of arthropods [28]. Chitinases break down chitin by hydrolyzing the β(1–4) linkages between N-acetylglucosamines [29]. Due to their mode of action, chitinases have been considered for their potential use in the control of arthropod pests [30,31,32]. Entomopathogenic fungi use chitinases as virulence and invasive factors to damage arthropods from the outside, and most entomopathogenic biocontrol fungi, such as *Metarhizium anisopliae* [30], *Beauveria bassiana* [31,33], *Isaria fumosorosea* [34], and those within the *Aspergillus* genus [20,35], exhibit abundant chitinase activity.

Aflatoxins are secondary metabolites usually produced by fungi within the *Aspergillus* genus and include approximately 20 toxic organic compounds. Among the more studied are B1, B2, G1, and G2, with B1 being more toxic, and the toxicity level comparison is considered to be B1 > G1 > B2 > G2 [36]. Aflatoxins are considered part of the human carcinogenic risk group 1 by the International Agency for Research on Cancer (IARC/WHO) because of their toxicity, bioaccumulation and thermostability. Aflatoxins bind to DNA and cause the transversion from guanine to thymine, causing liver cancer [37]. However, aflatoxins can also bind to proteins and sanguine albumin, causing systemic aflatoxicosis [38]. The aflatoxin synthesis gene cluster contains 25 to 30 genes and is approximately 70 kb in length [39,40]. The aflatoxin B1 biosynthesis pathway is a complex process involving at least 27 enzymatic reactions [41]. Aflatoxins are known to confer defense advantages against fungivores such as the fruit fly *Drosophila melanogaster* [42].

Kojic acid is a natural metabolite produced by fungi that inhibits melanin synthesis by blocking tyrosinase [43]. Kojic acid exhibits insecticidal properties when used on insects such as the milkweed bug *Oncopeltus spp.* and the house fly *Musca domestica* [44]. It has been patented as an insecticide synergist when used in combination with pyrethroid and carbamate formulations on the corn earworm *Helicoverpa zea* and the armyworm *Spodoptera frugiperda* [45]. It is also used in the cosmetic industry to lighten skin color and treat anomalies in skin pigmentation [46], as well as antibacterial compounds in the food industry, among many other uses [47]. 

Our study describes a new *A. flavus* strain isolated from naturally infected cattle ticks that was submitted to morphometric and biochemical analysis, as well as complete genome sequencing, comparative genomics, phylogenetic analysis, and bioassays. It is the goal of this work to study the effect of the fungus on ticks and describe the gene-coding entomopathogenic virulence factors found in this particular *A. flavus* isolate.

## 2. Materials and Methods

### 2.1. Isolation

A group of experimentally raised engorged ticks from the Media Joya strain [48] was obtained using the methodology previously described by Miranda-Miranda et al. [18]. Some of the collected ticks had natural and recurrent fungal infections. The ticks infected with fungi were identified by their dark cuticle color and the presence of fungal mycelium and conidiophore growth. The spores were collected and cultured on Sabouraud agar in Petri dishes, which were then incubated at 28 °C for three days. As a result of this process, a fungus labeled INIFAP-2021 was isolated, which is now a part of the strain collection of the Centro Nacional de Investigación Disciplinaria en Salud Animal e Inocuidad. This isolate has been previously reported by Arreguin-Perez et al. [49].

### 2.2. Microscopy Identification

The mycelium obtained from the Petri dishes was cultured on microscope glass slides covered with a thin layer of Sabouraud agar, following a method previously described by Miranda-Miranda et al. [18]. The cultures were then incubated at 28 °C for three days. Afterward, the fungal morphology and the cultures on microscope glass slides were identified using differential interference contrast microscopy (Axiovert 40 CFL, Carl Zeiss, Göttingen, Germany).

### 2.3. Morphotype Identification

To accurately determine the diameter of the sclerotium morphotype, 2 × 10^5^ conidia from an *A. flavus* isolate were evenly inoculated onto Petri dishes containing yeast extract glucose-tryptone agar (YGT). The dishes were then incubated at 28 °C for six days. Afterward, the sclerotia were harvested using a solution of 0.01% Triton X-100 (SIGMA, Saint Louis, MO, USA) in water, following a method previously reported by Gilbert et al. [14]. Fifty sclerotia were selected, and their measurements were conducted under a compound microscope at 40× magnification utilizing ImageJ (1.54f, Bethesda, MD, USA) software [50]. 

### 2.4. Genomic Comparison

An average nucleotide identity (ANI) comparison was performed using fastANI (v 1.34, USA) [51] between *A. flavus* INIFAP-2021 and the following *Aspergillus flavus* reference genomes: AF36 (GCA_012897275.1), K49 (GCA_012896705.1), NRRL3357 (GCA_014117465.1), AF70 (GCA_003711385.1), AZS04M2A (GCA_003711355.1, SU-16 (GCA_009856665.1), AF13 (GCA_014 117485.1), BS01 (GCA_003711305.1), DV901 (GCA_003711315.1), MC04 (GCA_003711285.1), *A. sojae* SMF134 (GCA_008274985.1), and *A. oryzae KJJ4b* (GCA_015.14).

### 2.5. Aflatoxin Identification

The INIFAP-2021 isolate was cultured on a Petri dish containing YGT for 6 days at 28 °C. Following this, ~25 mL of the fungus culture present on the agar was transferred to an assay tube, which contained three volumes of chloroform and two volumes of distilled water, and was disrupted by vigorous vortexing for 5 min, following a method previously reported by Yabe et al. abe da et al. [52]. Subsequently, the chloroform extract was moved to a new crystal tube, dried under vacuum conditions, and then solubilized in 5 mL of benzene-acetonitrile (98:2), as per a previously described procedure [52]. Analysis of the extract was conducted by the Centro Nacional de Servicios de Constatación en Salud Animal, Mexico, using high-performance liquid chromatography. The obtained results were compared to laboratory reference aflatoxin standards (SIGMA Laramie, WY, USA) [53], and the outcome of this experiment is presented in Table 1.

### 2.6. Chitosan-Based Medium Growth

According to a previous report by Miranda-Miranda et al. [18], a culture medium was prepared by combining phosphate-buffered saline (pH 7.2) with 10 g/L casein peptone and 1% chitosan. The medium was then sterilized and inoculated with INIFAP-2021 *A. flavus* spores. Subsequently, the inoculated culture was incubated at a temperature of 25 °C for 6 days under constant agitation at 60 rpm.

### 2.7. Spore Harvest

The INIFAP-2021 isolate was cultured on a Petri dish containing potato dextrose agar (PDA) and incubated at a temperature of 28 °C for 6 days. To harvest the spores, a modified method combining the techniques of Frerichis et al. [54], Gilbert et al. [14], and Shen et al. [55] was used. First, a sterile crystal triangle, previously immersed in a solution of 0.01% Triton X-100 in phosphate-buffered saline (PBS), was employed to gently scrape the spores attached to the glass. These spores were then rinsed with sterile distilled water, and the resulting spore–water suspension was filtered through #4 Whatman filter paper. Subsequently, the fungal spores present within the filtrate were resuspended in 5 mL of PBS and quantified using a Neubauer chamber, and the concentration was adjusted to 10^4^, 10^5^, 10^6^, and 10^7^ spores/mL in 10 mL aliquots.

### 2.8. Bioassay

The statistical design of this experiment consisted of 4 replicates with 10 ticks per experimental unit, utilizing a total of 200 acaricide-susceptible (Su) ticks from the Media Joya strain [48,56]. The engorged females were subjected to a washing process involving 100 mL of an aqueous solution containing 10% benzal, followed by two additional washes using 100 mL of distilled water for 10 min. The ticks were weighed after washing, following the adult immersion test previously described [57]. 

For treatment, four groups of ticks were submerged in 10 mL solutions containing 10^5^, 10^6^, 10^7^, and 10^8^ spores/mL for 10 min. An additional control group was submerged in PBS without fungal spores for the same timeframe and with the same statistical design as treatments. Notably, the control group included four replicas. After treatment, the ticks were dried using paper towels and individually transferred to wells in a 12-well culture plate.

After an incubation period of 15 days at 28 °C and 80% relative humidity, various parameters, including oviposition, mortality, morbidity, egg production index (EPI%), inhibition of oviposition (IO%), larval hatching inhibition percentage (IH%), and reproduction estimated inhibition (REI), were calculated for all groups using a method previously described by Drummond et al. [57]. Morbidity was limited to ticks with visible micellar growth. The equations for these parameters are as follows:

Mortality = (Dead females/Total females) × 100

Morbidity = (Sick females/Total females) × 100

EPI% = (Egg mass/Female initial mass) × 100

IO% = ((EPI% Control − EPI% Treatment)/EPI% Control) × 100

EC% = (Hatched larvae/(Hatched larvae + Unhatched eggs)) × 100

IH% = ((EC% Control − EC% Treatment)/EC% Control) × 100

RE = (EPI%/100) × (EC%/100) × 20000

REI = ((RE Control − RE Treatment)/RE Control) × 100

The analysis was conducted using R (4.05, R Core Team, Vienna, Austria) [58] and involved performing multiple one-way ANOVAs to assess the impact of different spore concentrations (10^5^, 10^6^, 10^7^, and 10^8^ spores/mL) on the oviposition, mortality, morbidity, EPI%, IO%, IH%, and REI. Furthermore, Tukey’s HSD test was applied to conduct multiple comparisons, with a confidence interval of 95%.

### 2.9. Virulence Factor Search

A comprehensive approach was employed to search for virulence factors in the *A. flavus* INIFAP-2021 complete genome assembly. This approach involved a combination of methods, including a thorough analysis of the genome assembly itself and a heuristic search based on relevant literature references, focusing on virulence factors in *Aspergillus* species. Specifically, the search targeted gene clusters involved in the synthesis of aflatoxins, kojic acid, ribotoxins, and chitinases. To identify these gene clusters, comparisons were made against the GenBank database using the BLAST toolkit. Additionally, the aflatoxin gene cluster from *A. parasiticus* (AY371490.1) [41] and the aflatoxin cluster of the closest reference genome (AF13) were utilized as reference sequences. The synteny between these clusters and the complete aflatoxin cluster was assessed by generating a synteny graph using SimpleSynteny [59]. The kojic acid biosynthesis gene cluster (Q2U5H8.1) from the *A. oryzae* RIB40 strain was referenced [60], along with the ribotoxin (KAB8244990.1) from *A. flavus*. Other secondary metabolites were identified using the antiSMASH fungal version [61]. Additionally, chitinases were identified by conducting a search within the gene ontology sequencing annotation of the *A. flavus* INIFAP-2021 strain [49].

**Table 1:** Comparison of genomes according to average nucleotide identity.

Query	R. Genome	ANI%	Af	Isolation Source	References
INIFAP2021	*A. sojae* SMF134	94.1089	No	Korean soybean fermented brick	[62]
INIFAP2021	*A. flavus* af70	98.9738	Yes	Soil	[14]
INIFAP2021	*A. flavus* azs04m2a	98.9784	Yes	Soil	[63]
INIFAP2021	*A. flavus* su-16	99.0755	No	*Huangjiu* fermenting starter	[64]
INIFAP2021	*A. oryzae* KJJ4b	99.0882	ND	Korean fermenting starter	[65] *
INIFAP2021	*A. flavus* BS01	99.196	Yes	Cotton seed	[15]
INIFAP2021	*A. flavus* MC04	99.2035	Yes	Cotton seed	[15]
INIFAP2021	*A. flavus* af36	99.204	No	Cotton	[66]
INIFAP2021	*A. flavus* k49	99.2076	No	Corn	[67]
INIFAP2021	*A. flavus* af13	99.277	Yes	Soil and corn	[13]
INIFAP2021	*A. flavus* DV901	99.2807	Yes	Cotton seed	[15]
INIFAP2021	*A. flavus* NRRL3357	99.5411	Yes	Peanut	[68]

R. genome = reference genome; Af = aflatoxin production; * Unpublished; ND: no data.

## 3. Results

### 3.1. Fungal Morphological Characterization

**Figure 1:**
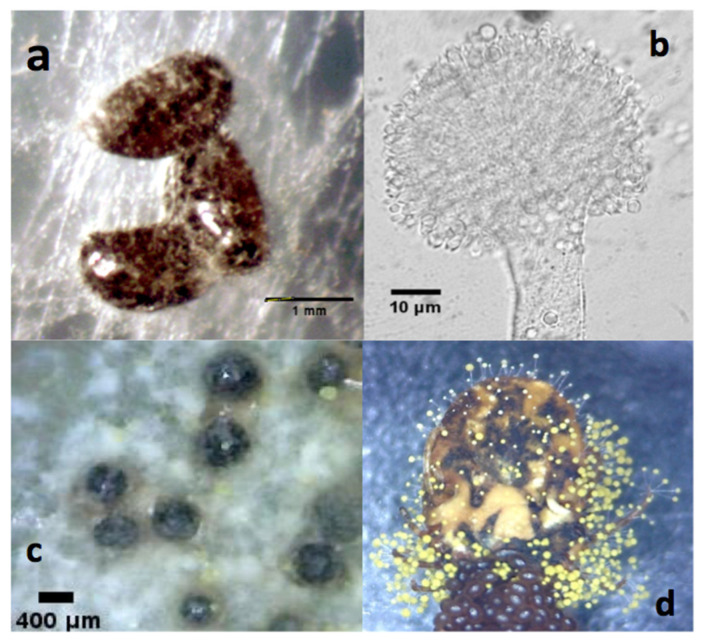
Identification of fungal infection on *R. microplus*. (**a**) Eggs exhibiting evident symptoms of fungal infection. (**b**) Microscopic characteristics of conidiophores. (**c**). Sclerotia from the isolated fungus. (**d**) Experimentally infected engorged female.

**Figure 2:**
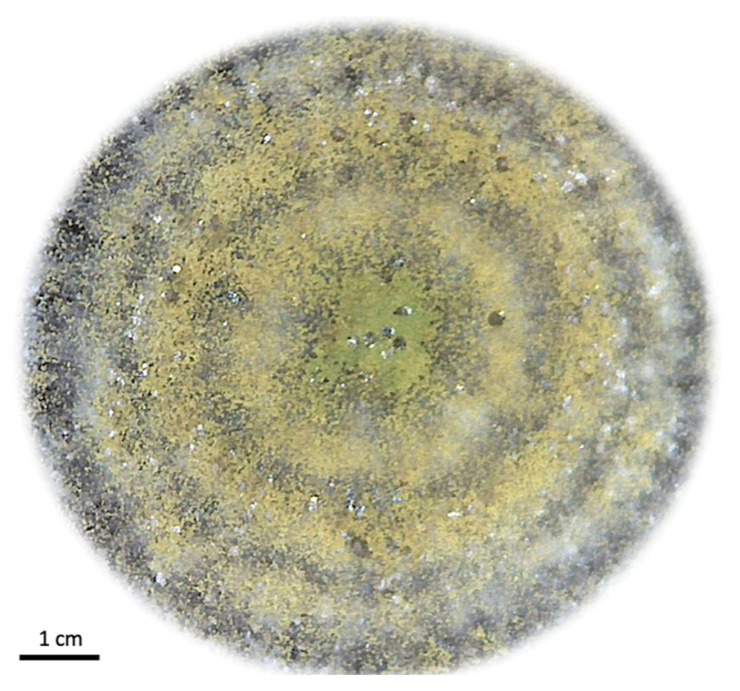
*A. flavus* INIFAP-2021 cultured on PDA. The fungal isolate was propagated on PDA plates exhibiting brownish-green filamentous colonies.

The *Aspergillus flavus* strain INIFAP-2021, isolated from naturally fungus-infected ticks [49], exhibited noticeable growth of mycelium and conidiophores on the tick cuticle (Figure 1d). These fungal spores obtained from the strain were used for isolation and propagation on SDA to evaluate the microscopic morphometric characteristics of the cultured isolate, and the colony was ~62 mm in diameter (Figure 2). This strain displayed septate and macro-siphoned hyphae, along with subglobose conidiophores and round spores (Figure 1b). The average diameter of the sclerotia was 418.82 µm (Figure 1c). Upon infection of *R. microplus* females, colonization of the cuticle was evident on the *alloscutum*, *scutum*, and *arthros*, accompanied by the presence of brownish-green mycelium and conidiophores (Figure 1d). Additionally, a more detailed examination of the infected engorged ticks revealed desiccation and a reduction in gut peristalsis.

### 3.2. Genomic Comparison

The results of the comparison with fastANI are shown in Table 1. This analysis shows that the closest genetic relationships of INIFAP-2021 are with the reference genomes NRRL3357, DV901, and AF13.

### 3.3. Aflatoxin Determination

The HPLC experiment for aflatoxin detection revealed the presence of aflatoxin B1 (Table 2) at a concentration of 424.2 mg/kg, 58.8 times higher than the concentration of aflatoxin B2 (7.2 mg/kg). Neither G1 nor G2 aflatoxins were detected.

### 3.4. Chitin-Based Medium Growth

The isolate *A. flavus* INIFAP-2021 was capable of propagating, exhibiting visible mycelium when using chitosan as the sole source of carbon.

### 3.5. Bioassay Results

The entomopathogenic fungus *A. flavus* strain INIFAP-2021 was assessed for its effects on engorged *R. microplus* females through immersion in varying concentrations of spore-containing aqueous solutions. The effects measured included mortality, morbidity, egg production index, inhibition of oviposition, hatching, inhibition of larval hatching, and estimated reproduction inhibition. The results of these assessments are summarized in Table 3.

Statistical analysis was performed using R and R Studio (1.41106, PBC, Boston, MA, USA) and indicated that the treatments exhibited a significant effect on mortality (F(4,15) = 11.37, *p* = 0.0002; t(15) = 2.131, *p* < 0.05) when the ticks were submerged in a solution containing 10^7^ spores/mL; on morbidity (F(4,15) = 230.4, *p* < 0.0001; t(15) = 2.131, *p* < 0.05) at 10^5^ spores/mL; on EPI%, there was no significant effect (F(4,15) = 2.922, *p* = 0.569), and on larval hatching inhibition (F(4,195) = 3.252, *p* = 0.0131; t(195) = 1.962, *p* < 0.05) at 10^8^ spores/mL. The summarized results can be found in Table 3.

### 3.6. Virulence Factor Search

#### 3.6.1. Aflatoxins

**Figure 3:**
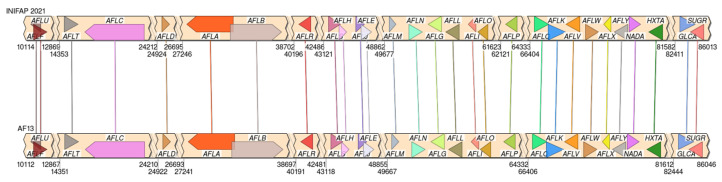
Synteny between INIFAP 2021 (Above) and AF13 (AY510451.1) (Below) aflatoxin clusters using *A. parasiticus* aflatoxin gene cluster (AY371490.1) as query.

**Figure 4:**
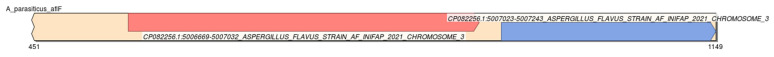
Synteny between the incomplete aflF gene from INIFAP 2021 and aflF from *A. parasiticus* (AY371490.1).

Comparative genomics analysis revealed the presence of the complete aflatoxin biosynthesis cluster in the *A. flavus* INIFAP-2021 genome, spanning 88,285 bp with a total of 29 genes (Table 4). The cluster exhibited an average coverage of 97.10%, an average E value of 1.035 × 10^−147^, and an average identity of 96.02%. Furthermore, the synteny evaluation demonstrated a sequence correlation between this cluster and the one located on chromosome 3 of the reference genome CP082256.1 (Figure 3). Additionally, we analyzed the aflF gene, which was found to be fragmented and incomplete (Figure 4). The total length of the *A. parasiticus* aflF gene (AY371490.1) was 1149 bp, whereas the INIFAP 2021 fragments of the aflF gene had lengths of only 364 and 221 bp. It is important to note that these fragments overlapped by nine base pairs within the genome. Figure 4 illustrates how these fragments align with the reference aflF gene. Moreover, the gene is missing 574 bp at the beginning, including the active site, which should typically be located at around position 207 of the gene. The synteny were visualized using SimpleSynteny (v1.6, Beltsville, MD, USA). 

#### 3.6.2. Kojic Acid

**Figure 5:**
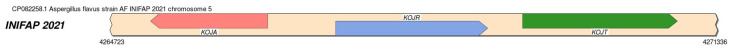
Synteny between INIFAP 2021 and *A. oryzae* RIB40 (XM_001824266.1, XM_001824267.1, and XM_001824268.1) kojic acid clusters.

The results of comparative genomics analysis comparing the INIFAP-2021 genome in chromosome 5 CP082258.1 to the reference RIB40 kojic cluster (XM_001824266.1, XM_001824267.1, and XM_001824268.1) revealed the presence of the kojic acid biosynthesis gene cluster on chromosome 5, with an average coverage of 100%, an E value of 0.0, and an identity of 99.9% (Table 5). To assess the synteny of the complete kojic acid cluster, a synteny graph was generated using SimpleSynteny (Figure 5), confirming the presence of the complete and likely functional gene cluster in the genome [59]. 

#### 3.6.3. Ribotoxin

A ribotoxin coding gene was found on chromosome 2 when compared to the reference genome CP082255.1, exhibiting a total score, query coverage, E value, and identity of 100% coverage and 85.62% identity, as summarized in Table 5.

#### 3.6.4. Chitinases

Bioinformatic analysis of the genome revealed the presence of chitinase-encoding genes with similarity to those from fungal genera such as *Rhizopus*, *Arthroderma*, *Aphanocladium*, *Streptomyces,* and *Aspergillus,* as well as *Aspergillus* teleomorphs such as *Emericella* and *Neosartorya* [69]. The predicted genes are summarized in Table 6 and are all predicted to express exo- and endochitinases.

#### 3.6.5. No Expected Virulence Factors 

A thorough search using the AntiFungi algorithm revealed the noteworthy secondary metabolite napthopyrone, a fungal predator-protecting secondary metabolite [70] (Table 7). Moreover, comparative genomics results revealed the presence of toxin-related genes, comprising those related to killer toxin α/β, satratoxin, and aflatoxins identified in fungal genera such as *Kluyveromyces*, *Stachybotrys*, *Aspergillus*, the teleomorph genus *Neosartorya*, and the KP4 killer toxin from *Ustilago maydis* P4 virus [10,71,72] (Table 8).

## 4. Discussion

The aim of this work was to isolate, identify, and characterize the fungal infection of cattle ticks, for which we isolated *A. flavus* from the various developmental stages of ticks, such as eggs, larvae, and adults, as previously reported [18]. We obtained a new strain of *A. flavus* from the same location as the previously reported strain, indicating the persistent presence of *A. flavus* in the habitat over the years. Our morphological analysis was consistent with that of *A. flavus*, exhibiting septate and macro-siphoned hifae, globose conidiophores, green spores, and the production of sclerotia [14,15,18]. The differential analysis of the *A. flavus* isolate INIFAP-2021 revealed that it is closely related to L morphotype *A. flavus* [15], exhibiting an average sclerotia size of 418.82 µm and only producing aflatoxins B1 and trace amounts of B2, in contrast to the four usually found in *A. flavus* (B1, B2, G1, G2) [39]. This difference can be attributed to the only two genes with less than 90% coverage in comparison to the reference cluster, aflU and aflF, both of which are essential for the production of G-type aflatoxins [83]. The incomplete aflF gene synteny is displayed in Figure 4. This isolate is different from the cattle tick-infecting *A. flavus* previously reported, which is capable of synthesizing B1 together with GI and G2 [18]. The *A. flavus* INIFAP 2021 strain was found to exclusively produce aflatoxin type B. 

Fungal infection bioassays displayed a morbidity rate of 90% at a concentration of 10^5^ conidia/mL and a mortality rate of 50%, with an oviposition inhibition of 21.5% at 10^7^ conidia/mL. At a concentration of 10^8^ conidia/mL, a larval hatch inhibition of 30.8% and an estimated reproduction inhibition of 34.64% were observed. Furthermore, compared to the uninfected control (Table 1), this isolate showed a significantly lower egg production index. The oviposition of treatment 10^7^ showed high variability, with one replica exhibiting a very low oviposition rate, thereby affecting oviposition inhibition (IO%), as indicated by its standard deviation. It is important to note that oviposition is an independent parameter from both hatching percentage and hatching inhibition (IH%), and therefore, they may not necessarily be correlated when oviposition is present. It is also worth noting that this strain is distinct from the one previously reported by Miranda-Miranda et al. [18], which demonstrated a mortality rate of 64% in engorged cattle tick females during bioassays and the production of aflatoxin types B and G. Other entomopathogenic fungal genera, such as *Beauveria bassiana* and *Metarhizium anisopliae*, have also exhibited mortality effects ranging from 80 to 90% and 90 to 100%, respectively, at concentrations from 1 × 10^6^ to 1 × 10^9^ spores/mL and 1 × 10^6^ to 1 × 10^8^ spores/mL, respectively [84,85,86]. However, the INIFAP 2021 isolate is worth studying due to the novelty of its infection and the already-known industrial growth conditions [87]. Therefore, this strain holds promise for potential biocontrol against *R. microplus*.

Killer toxins from *Kluyveromyces* can arrest proliferation, and the α and β subunits have exochitinase activity [71]. Moreover, growth in a chitosan-based medium reveals the isolate’s capability of using chitosan as the sole carbon source, indicating the presence of chitinases. Chitinases may play a role in infection processes, such as degrading the cuticle, as observed in *Beauveria bassiana* [88] and *Metarhizium anisopliae* [30,89]. Killer toxins from *Kluyveromyces* possess α and β subunits with exochitinase activity [71], whereas the complete genome sequence of *A. flavus* INIFAP-2021 revealed at least nine predicted chitinases, providing evidence that these molecules function as virulence factors in multiple genera, such as *Metarhizium* and *Beauveria* (Table 5).

Further investigation is needed to corroborate the importance of possible virulence factors and their role in infection, including the efficacy of kojic acid against *R. microplus*. Kojic acid has been used as an insecticide [45] and a development inhibitor of *Drosophila melanogaster* [90], but no information is available on its use as an acaricide. Thus, assessing the effectiveness of kojic acid against *R. microplus* is essential.

Efficient conversion from glucose to kojic acid in a glucose-citrate buffer medium has been achieved without the need for microbial growth [91]. Furthermore, the presence of chitinases and the ability of INIFAP 2021 to grow with chitosan as its sole carbon source suggests that chitin could be metabolized by this organism, and we propose a plausible metabolic pathway from chitin to kojic acid. This pathway involves the following reactions: chitin → chitobiose → N-acetyl-D-glucosamine → N-acetyl-D-glucosamine 6 phosphate → D-glucosamine 6 phosphate → beta-D-fructose 6-phosphate → glucose by gluconeogenesis → oxykojic acid → kojic acid. The first seven steps of this pathway are taken from the KEGG ko00520 pathway, whereas the last two steps have already been reported [92]. However, more investigation and experiments are needed to verify the existence of this pathway.

Despite the existing knowledge of the disruption of the sarcin-ricin loop by ribotoxins, which leads to inhibited protein synthesis and host death [23,25,93], and the known presence of these toxins in biocontrol microorganisms such as *Hirsutella* and *Metarhizium* [24,94], there is still much unknown information about these potential virulence factors. This includes their regulation and contribution to the infection of *R. microplus*. To gain a better understanding of the importance of these predicted molecules as virulence factors, it is necessary to directly investigate the potential production of secondary metabolites and toxin-related genes predicted in INIFAP-2021 by Augustus and AntiFungi, as shown in Table 7 and Table 8.

## 5. Conclusions

The cattle tick naturally infected with the fungus isolate INIFAP-2021, which was identified as *A. flavus* and classified as morphotype L, may effectively affect *R. microplus* development due to numerous virulence factors during *A. flavus* infection, such as the gene clusters dedicated to the biosynthesis of secondary metabolites in its genome. Experimental bioassays revealed this isolate to be lethal to cattle ticks, producing mortality and morbidity and reducing larval hatching, with 1 × 10^8^ spores/mL being the most effective concentration. This organism exhibits promising potential as an entomopathogenic fungus, either as a complete organism or through its virulence factors. However, further studies are necessary to enhance treatment efficiency, such as by exploring the use of adjuvants, and to investigate host specificity. 

## Figures and Tables

**Table 2:** Aflatoxin identification and concentration of benzene-acetonitrile (98:2) extract from *A. flavus* INIFAP 2021.

Aflatoxin Type	Concentration (mg/kg)
B1	424.2
B2	7.2
G1	ND
G2	ND

ND means not detected.

**Table 3:** Averages and standard deviations of mortality, morbidity, egg production index (EPI%), inhibition of oviposition (IO%), hatching (H%), inhibition of larval hatching (IH%), and estimated reproduction inhibition (ERI%).

Conidia/mL	Mort.%	Morb.%	EPI%	IO%	H%	IH%	ERI%
Control	5 (± 10) ^a^	2.5 (±5) ^a^	53.4 (±2.0) ^NS^	0 (±12.0) ^NS^	85.6 (±9.2) ^a^	0 (±11.3) ^a^	0 (±23.3) ^NS^
10^5^	10 (±14.1) ^a^	90 (±11.5) ^b^	50.7 (±5.7) ^NS^	0.08 (±11.3) ^NS^	75.7 (±16.9) ^ab^	7.0 (±20.1) ^ab^	8.68 (±20.0) ^NS^
10^6^	15 (±5.8) ^a^	100 (0) ^b^	53.4 (±6.8) ^NS^	0.95 (±11.2) ^NS^	70.6 (±7.3) ^ab^	13.2 (±9.0) ^ab^	12.88 (±14.5) ^NS^
10^7^	50 (±14.1) ^b^	100 (0) ^b^	39.8 (±11.2) ^NS^	21.5 (±22.1) ^NS^	67.5 (±14.8) ^ab^	17.0 (±18.2) ^ab^	32.95 (±29.0) ^NS^
10^8^	50 (±18.3) ^b^	100 (0) ^b^	48.6 (±2.5) ^NS^	4.27 (±4.9) ^NS^	56.3 (±8.6) ^b^	30.8 (±11.3) ^b^	34.63 (±8.1) ^NS^

^NS^ means no significant differences; letters indicate significant differences (Tukey, *p* < 0.05); Mort. = mortality; Morb. = Morbilitiy.

**Table 4:** Blast data from aflatoxin cluster of *A. parasiticus* (AY371490.1) vs. INIFAP-2021.

Gene	Max Score	Total Score	Cover (%)	E. Value	Identity (%)
*aflF*	529	866	50	2.00 × 10^−149^	92.96
*aflU*	1360	2109	86	0.0	95.94
*aflT*	1127	2778	99	0.0	99.36
*aflC*	6689	11,389	100	0.0	99.09
*aflD*	942	1431	100	0.0	98.15
*aflA*	8100	8887	100	0.0	98.71
*aflB*	8248	10,065	100	0.0	98.56
*aflR*	2338	2338	100	0.0	98.28
*aflS*	1070	2357	100	0.0	98.83
*aflH*	1480	1480	100	0.0	98.57
*aflJ*	1567	2058	96	0.0	97.59
*aflE*	1005	1005	100	0.0	98.42
*aflM*	518	1251	99	3.00 × 10^−146^	95.12
*aflN*	1513	2159	100	0.0	93.99
*aflG*	1777	2409	99	0.0	95.98
*aflL*	1879	2487	100	0.0	96.09
*aflI*	1199	1199	98	0.0	92.23
*aflO*	1216	1888	100	0.0	95.76
*aflP*	640	2145	100	0.0	98.61
*aflQ*	608	2484	100	3.00 × 10^−173^	94.03
*aflK*	2152	3010	100	0.0	94.31
*aflV*	1947	2382	93	0.0	96.9
*aflW*	2121	2121	98	0.0	93.43
*aflX*	1105	1105	99	0.0	91.61
*nadA*	1158	1758	99	0.0	90.07
*htxtA*	1158	2716	100	0.0	98.19
*glcA*	1491	2905	100	0.0	96.77
*sugR*	2008	2345	100	0.0	94.87

**Table 5:** Blast data from the kojic acid cluster of *A. oryzae* RIB40 vs. INIFAP-2021 and ribotoxin from *A. flavus* vs. INIFAP-2021.

Gene	Max Score	Total Score	Cover (%)	E. Value	Identity (%)
*kojA*	2377	2377	100	0.0	100
*kojR*	3070	3070	100	0.0	99.88
*kojT*	3114	3114	100	0.0	99.82
*Ribotoxin*	285	361	100	3 × 10^−89^	85.85

**Table 6:** Chitinase-related genes predicted in INIFAP-2021 by Augustus.

Code	Name	Similarity (%)	Organism Source
g1259.t1	Endochitinase	51.12	*Emericella nidulans*
g1746.t1	Chitinase	43.4	*Rhizopus oligosporus*
g2988.t1	Endochitinase	66.5	*Neosartorya fumigata*
g3174.t1	Endochitinase	74.52	*Emericella nidulans*
g3945.t1	Endochitinase	73.19	*Neosartorya fumigata*
g6394.t1	Endochitinase	66.65	*Aspergillus niger*
g6415.t1	Class III chitinase ARB_03514	53.23	*Arthroderma benhamiae*
g8743.t1	Class III chitinase ARB_03514	55.92	*Arthroderma benhamiae*
g8762.t1	Chitinase 1	45.25	*Aphanocladium album*

**Table 7:** Secondary metabolite regions identified by Antifungi; only >40% similarity clusters are shown.

Chromosome	Most Similar Known Cluster	Similarity (%)	Secondary Metabolite	References
CP082254.1	Asparasone A	75	Pigment for sclerotia	[73]
CP082255.1	Monascorubrin	100	Red pigment	[74]
	Fusarin	100	Polyketide	[75]
CP082256.1	Cyclopiazonic acid	71	Inhibition of reticulum calcium-dependent ATPase	[76]
CP082257.1	Clavaric acid	100	Antitumoral	[77]
	Naphthopyrone	100	Antibiotic building block, predator protection	[70]
CP082258.1	Pyranonigrin E	100	Antioxidant	[78]
CP082259.1	Clavaric acid	100	Antitumoral	[77]
	Penicilin	63	Antibiotic	[79]
	Aspirochlorine	54	Inhibitor of fungal protein synthesis	[80]
CP082260.1	6-methylsalicyclic acid	100	Antibiotic building block	[81]
CP082261.1	Squalestin S1	40	Squalene synthetase inhibitor	[82]

**Table 8:** Toxin-related genes predicted in INIFAP-2021 by Augustus.

Code	Name	Similarity (%)	Organism Source
g8.t1	Killer toxin subunits alpha/beta	46.49	*Kluyveromyces lactis*
g3204.t1	KP4 killer toxin	47.37	*Ustilago maydis P4 virus*
g3205.t1	KP4 killer toxin	46.38	*Ustilago maydis P4 virus*
g5455.t1	Aflatoxin cluster transcriptional coactivator aflS	58.56	*Aspergillus parasiticus*
g5456.t1	Aflatoxin biosynthesis regulatory protein	51.67	*Aspergillus flavus*
g5520.t1	Killer toxin subunits alpha/beta	43.52	*Kluyveromyces lactis*
g7698.t1	Satratoxin biosynthesis SC1 cluster transcription factor SAT9	43.25	*Stachybotrys chartarum*
g8303.t1	Satratoxin biosynthesis SC1 cluster protein 4	46.15	*Stachybotrys chartarum*
g9654.t1	MFS gliotoxin efflux transporter gliA	62.29	*Neosartorya fumigata*
g9945.t1	Toxin subunit YenA2	46.51	*Yersinia entomophaga*
g10528.t1	MFS gliotoxin efflux transporter gliA	58.19	*Neosartorya fumigata*
g10725.t1	Killer toxin subunits alpha/beta	44.19	*Kluyveromyces lactis*
g10752.t1	Satratoxin biosynthesis SC1 cluster protein 4	43.16	*Stachybotrys chartarum*

## Data Availability

The data presented in this study are openly available at https://www.ncbi.nlm.nih.gov/assembly/GCA_019880445.1/#/def, accessed on 5 September 2021 https://www.ncbi.nlm.nih.gov/bioproject/PRJNA758689/, accessed on 5 September 2021.
